# Conserved MicroRNAs in Human Nasopharynx Tissue Samples from Swabs Are Differentially Expressed in Response to SARS-CoV-2

**DOI:** 10.3390/genes13020348

**Published:** 2022-02-14

**Authors:** Ales Eichmeier, Tomas Kiss, Maria Kocanova, Eliska Hakalova, Milan Spetik, Jana Cechova, Boris Tichy

**Affiliations:** 1 Mendeleum—Institute of Genetics, Mendel University in Brno, Valticka 334, 691 44 Lednice, Czech Republic; tomas.kiss@mendelu.cz (T.K.); maria.kocanova@mendelu.cz (M.K.); eliska.hakalova@mendelu.cz (E.H.); milan.spetik@mendelu.cz (M.S.); jana.cechova@mendelu.cz (J.C.); 2 Center of Molecular Medicine, Central European Institute of Technology, Masaryk University, Kamenice 5, 625 00 Brno, Czech Republic; boris.tichy@ceitec.muni.cz

**Keywords:** miRNAs, small RNA sequencing, SARS-CoV-2, real-time RT-PCR, mir-21

## Abstract

The use of high-throughput small RNA sequencing is well established as a technique to unveil the miRNAs in various tissues. The miRNA profiles are different between infected and non-infected tissues. We compare the SARS-CoV-2 positive and SARS-CoV-2 negative RNA samples extracted from human nasopharynx tissue samples to show different miRNA profiles. We explored differentially expressed miRNAs in response to SARS-CoV-2 in the RNA extracted from nasopharynx tissues of 10 SARS-CoV-2-positive and 10 SARS-CoV-2-negative patients. miRNAs were identified by small RNA sequencing, and the expression levels of selected miRNAs were validated by real-time RT-PCR. We identified 943 conserved miRNAs, likely generated through posttranscriptional modifications. The identified miRNAs were expressed in both RNA groups, NegS and PosS: miR-148a, miR-21, miR-34c, miR-34b, and miR-342. The most differentially expressed miRNA was miR-21, which is likely closely linked to the presence of SARS-CoV-2 in nasopharynx tissues. Our results contribute to further understanding the role of miRNAs in SARS-CoV-2 pathogenesis, which may be crucial for understanding disease symptom development in humans.

## 1. Introduction

Severe acute respiratory syndrome coronavirus-2 (SARS-CoV-2) is currently a global threat leading to considerable disease and mortality worldwide. SARS-CoV-2 is a close relative of SARS-CoV with 45–90% sequence similarity and has resulted in more than 8000 confirmed cases of severe acute respiratory syndrome in [1,2]. Coronaviruses are a diverse family of viruses associated with multiple respiratory diseases with different severities, such as the common cold, pneumonia, and COVID-19 [3].

SARS-CoV-2 belongs to the order *Nidovirales*, family *Coronaviridae*, subfamily *Orthocoronavirinae*, genus *Betacoronavirus*, and subgenus *Sarbecovirus*. It has a single-stranded positive-sense RNA genome, 26–32 kilobases (kb) in length [4]. Similar to an mRNA, the virus genome consists of a 5′ cap structure together with a 3′ poly(A) tail that translates its proteins. Most of the genome at the 5′ end is occupied by the replicase gene, which encodes polyproteins ORF1a and ORF1b. ORF1a and ORF1b are further processed to generate nonstructural proteins (nsps). ORF1a contributes to the production of nsp1–nsp11, while the rest of the nsps (nsp12–nsp16) originate from ORF1b [5]. Additionally, viral structural proteins comprise surface (S), envelope (E), membrane (M), and nucleocapsid (N) proteins encoded by one-third of the genome at the 3′ end [5,6].

Small noncoding endogenous RNAs called microRNAs (miRNAs) play a major role in posttranscriptional gene regulation related to diverse biological processes, including development, immune system responses, or cell death [7]. The aspects of viral replication and proliferation that are included in host antiviral responses and viral pathogenesis may be influenced by miRNAs. A class of miRNAs function by directly binding to the target transcript. Ideal binding in the seed region has an important impact on the regulatory function of a miRNA. The seed sequence or seed region is a conserved heptametrical sequence. Even though base pairing between the miRNA and its target mRNA does not match perfectly, the seed sequence must be perfectly complementary [8]. miRNAs might hold a negative or positive role in virus-related processes in three ways: direct binding to the viral genome, binding to viral transcripts, or binding to host transcripts [8]. Human miRNAs can promote stability, replication, and infection of viral RNA, they also can reinforce host antiviral responses against viruses. miRNAs can also be used for antiviral therapeutic approaches [9,10,11].

Computational analyses of high-throughput sequencing data, followed by experimental validation, have been used to identify highly conserved miRNAs, some of which play important roles in human ontogeny [12,13,14,15]. In total, 2300 true human mature miRNAs were extrapolated, of which 1115 were annotated in miRBase V22 [16]. miRNA expression analysis could indicate links between gene expression regulation in human nasopharynx tissue and the response to SARS-CoV-2 infection and could also reveal which miRNAs undergo changes in expression in response to the infection.

We hypothesize that the obtained miRNA profiles will show differences between SARS-CoV-2-negative and SARS-CoV-2-positive RNA samples.

## 2. Materials and Methods

### 2.1. RNA Samples, RNA Extraction, and Real-Time RT-PCR Detection of SARS-CoV-2

Total RNA was extracted from the nasopharynx using flocked swabs and a 1 mL viral transport medium (various vendors). RNA was extracted using an in-house method adapted from He et al. [17] using Sera-Mag beads (GE Healthcare, Chicago, IL, USA). Real-time RT-PCR was performed according to the diagnostic detection of Wuhan coronavirus 2019 by real-time RT-PCR [18]. RdRp, E, and human RNAseP assays were run in separate real-time RT-PCR reactions using a GB OneStep IPC Elite real-time RT-PCR Kit (Generi Biotech, Hradec Kralove, Czech Republic), which employs an internal positive control for the detection of PCR inhibition. For relative quantification of the viral titer in positive samples, the E gene assay results were normalized to hsa-mir-148a housekeeping miRNA. The reverse transcription and real-time PCR conditions for the hsa-mir-148a assay were, except for the fact that the RNA was not diluted, the same as described below in the validation of miRNA expression profiles by real-time RT-PCR section. The relative quantity of viral titer was calculated according to the 2^−ΔΔC^t method [19] by using the qBase+ software (Biogazelle, Zwijnaarde, Belgium). In total, the study was conducted on 10 SARS-CoV-2-positive (PosS) and 10 SARS-CoV-2-negative (NegS) RNA samples listed in Table 1.

### 2.2. Small RNA Library Preparation and High-Throughput Sequencing

A small RNA library was constructed using the NEBNext^®^ Small RNA Library Prep Set for Illumina^®^ (NEB, Ipswich, MA, USA), and purification was performed with the TailorCut Gel Extraction Tool Set (SeqMatic, Fremont, CA, USA). The quality and quantity of the library were determined using the Agilent High Sensitivity DNA Kit (Agilent, Santa Clara, CA, USA). The quantity of the library was also determined by a Modulus™ Single Tube Multimode Reader (Turner Biosystems, Sunnyvale, CA, USA) using a Quant-iT™ dsDNA Assay Kit (Thermo Fisher Scientific, Waltham, MA, USA) and finally with an MCNext™ SYBR^®^ Fast qPCR Library Quantification Kit (MCLAB, San Francisco, CA, USA) used with Rotor-Gene 3000 (Corbett Research, Sydney, Australia). All kits were used according to the manufacturer’s instructions. The libraries were pooled at a concentration of 2 nM according to fluorimetry measurements, assuming that the final cloned small RNA products were ~150 bp.

For each sequencing run, a final pooled library of small RNAs consisted of pooled samples. In total, 20 RNA samples were sequenced, 10 SARS-CoV-2-negative and 10 positive RNA samples, which were previously tested by real-time PCR. In total, six runs were performed on a MiniSeq instrument (Illumina, San Diego, CA, USA). The MiniSeq High Output Reagent Kit, 75 cycles (Illumina, San Diego, CA, USA) providing 36-nt long reads, was used.

### 2.3. Bioinformatics and Data Evaluation

Sequence quality was controlled by using FastQC-0.10.1 [20]. Then, the reads were imported to CLC Genomics Workbench 6.5.1 (CLC Bio, Aarhus, Denmark) using the following parameters: sequence length 10–50 nucleotides, no ambiguous nucleotides, removal of the Illumina universal adapter sequence, and a Phred score assigned a Q score of 30 (Q30); reads were trimmed using the following parameters: removal of smallRNA_adapter (TGGAATTC), removal of sequences of length: minimum 19 nucleotides and maximum 25 nucleotides. The total number of known miRNAs was counted and annotated using miRbase—Release 22.1 (Homo sapiens) in CLC Genomics Workbench 6.5.1 (CLC Bio, Aarhus, Denmark). The statistical method to quantify differential expression in CLC Genomics Workbench 6.5.1 was used: Transcriptomic Analysis: Small RNA Analysis: Extract and Count and Annotate and Merge Counts. The numbers of miRNA sequences were normalized to 1 million reads (RPM) in order to enable a comparative analysis. Statistics as Kruskal–Wallis test, ANOVA, PCA, and scattered plot graphs were performed using PAST version 2.17c [21].

### 2.4. Validation of miRNA Expression Profiles by Real-Time RT-PCR

For validation of miRNA expression profiles, hsa-mir-21, hsa-mir-34b, hsa-mir-34c, and hsa-mir-342 and a housekeeping hsa-mir-148a were chosen. These miRNAs were selected according to the statistical significances of small RNA sequencing results. Prior to reverse transcription, the RNA samples were treated with DNase I (Sigma-Aldrich, Saint-Louis, MO, USA). From each sample, 1 ng of RNA was used for reverse transcription of each miRNA by using the TaqMan MicroRNA Reverse Transcription Kit Assay (Applied Biosystems, Foster City, CA, USA) according to the manufacturer’s instructions. Oligonucleotides for reverse transcription and real-time PCR of each miRNA were supplied in TaqMan MicroRNA Assays (Applied Biosystems, Foster City, CA, USA), where the assay IDs for tested miRNAs were as follows: ID: 000397 for hsa-mir-21; ID: 000427 for hsa-mir-34b; ID: 000428 for hsa-mir-34c; ID: 002147 for hsa-mir-342; ID: 000470 for hsa-mir-148a. Real-time PCR amplification conditions and reactions using TaqMan Universal Master Mix II (Applied Biosystems, Foster City, CA, USA) were performed according to the TaqMan MicroRNA Assay (Applied Biosystems, Foster City, CA, USA) instructions. Each miRNA sample was amplified in triplicate and run in a qTower cycler (Analytik Jena, Jena, Germany). Prior to miRNA expression measurement, the primer pair efficiency (E) values were evaluated on the standard curves of serial dilutions of pooled cDNA for each miRNA. For miRNA expression normalization, the ΔΔCt method [22] was used with hsa-mir-148a miRNA as a normalizer using qPCRsoft 3.4 software (Analytik Jena, Jena, Germany). The normalized expression values were then statistically evaluated by analysis of variance (ANOVA) (*p*-value ≤ 0.05) in Statistica 13 software (Tibco, Palo Alto, CA, USA). The same software was used in the analysis of correlation (Pearson’s correlation coefficient) between relative viral quantity and normalized miRNA expressions.

## 3. Results

### 3.1. The Abundance of miRNAs Detected in Nasopharynx Tissues

The selected samples from the SARS-CoV-2 negative group (NegS) were assayed by using the SARBECO primer detection protocol [18] and were determined to be negative based on a Ct value threshold of 40. Samples from the SARS-CoV-2-positive group (PosS) were assayed by the same real-time RT-PCR method and the Ct values ranged from 24.82 (G9) to 33.75 (F5). The median Ct of the PosS group was 30.71. After normalization, the relative viral quantities ranged in the PosS group between 2.2 (F5) to 18529.1 (A7) showing almost a 10,000× difference in the viral quantity within the samples (Table 1). The median relative viral quantity of the PosS group was 90.1.

In the present study, libraries representative of small RNA populations extracted from nasopharynx tissues and sequenced by Illumina SBS technology contained a quality of Q30 following the number of reads: in the NegS group ranging from 510,211 (3A) to 5,844,091 (13A) reads and in the PosS group ranging from 178,950 (D3) to 5,007,768 (H5) reads, the normalized numbers of reads are shown in Table 2. Sequencing data are deposited under BioProject acc. no. PRJNA747809, where the SRA experiments are available by acc. nos. SRX11490726–SRX11490735 (NegS group) and SRX11493797–SRX11493806 (PosS group).

**Figure 1:**
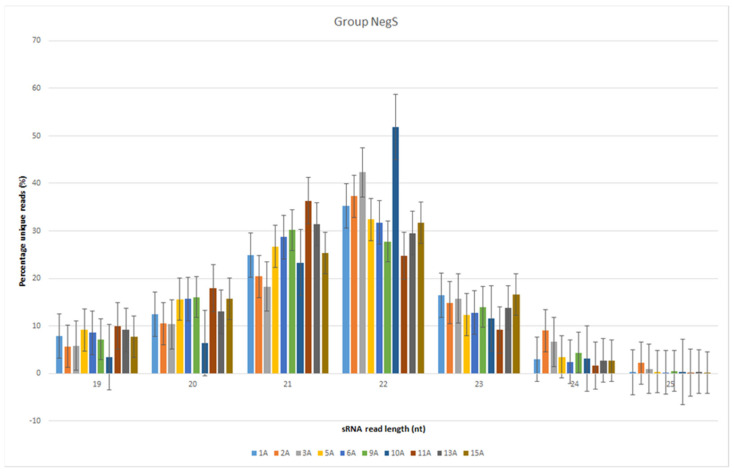
Bar plot depicting the size distribution of unique reads in the 19- to 25-nt class of sRNAs in the NegS group. Standard errors are highlighted by vertical error lines. Each sample is distinguished by a specific color.

**Figure 2:**
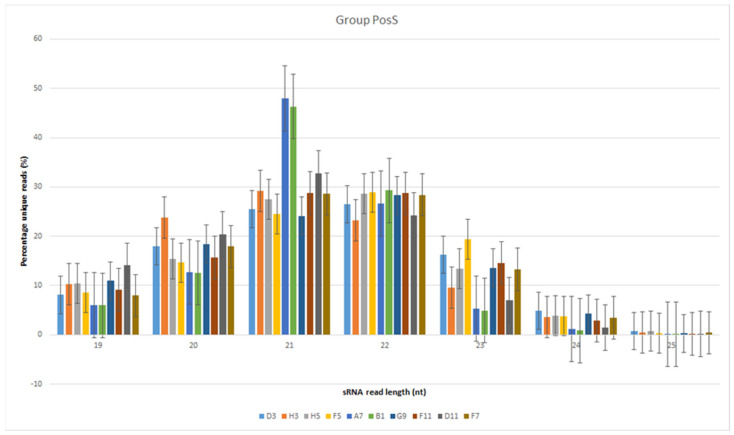
Bar plot depicting the size distribution of unique reads in the 19- to 25-nt class of sRNAs in the PosS group. Standard errors are highlighted by vertical error lines. Each sample is distinguished by a specific color.

The reads were normalized to reads per million values. The most abundant sRNAs belonged to the 21- and 22-nt classes in general (Figure 1 and Figure 2). The highest median read value was for the 21-nt class sRNAs in both the NegS (9,598 reads) and PosS (6331 reads) groups. However, the average value in the NegS group (Figure 1 and Figure 2) was the highest for the 22-nt class (13,261 reads), and that in the PosS group was the highest for the 21-nt class (15,088 reads) sRNAs (Table 2).

### 3.2. Conserved miRNAs Identified in Nasopharynx Tissues

**Figure 3:**
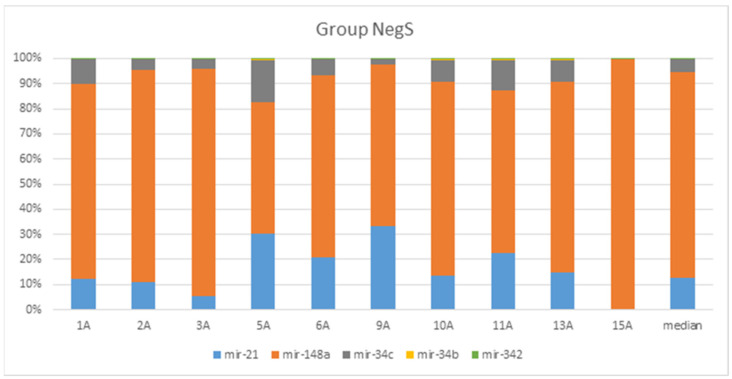
Bar plot depicting the proportions of five known selected human mature miRNAs according to their different abundances in both groups across NegS, CLC Genomics Workbench 6.5.1, and miRBase Release 22.1. Proportions were calculated based on normalized total reads.

**Figure 4:**
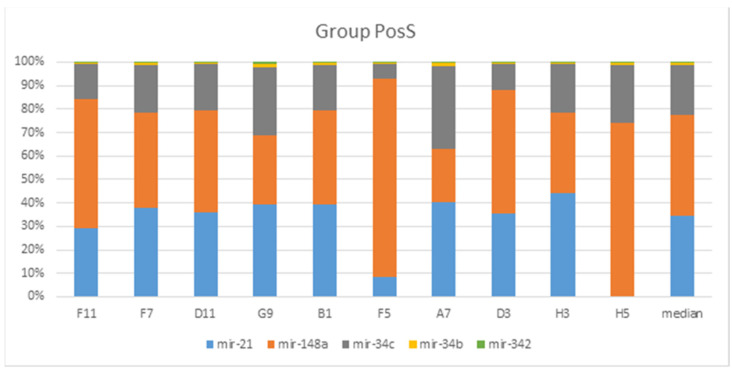
Bar plot depicting the proportions of known selected human mature miRNAs according to their different abundances in both groups across PosS, CLC Genomics Workbench 6.5.1, and miRBase Release 22.1. Proportions were calculated based on normalized total reads.

**Figure 5:**
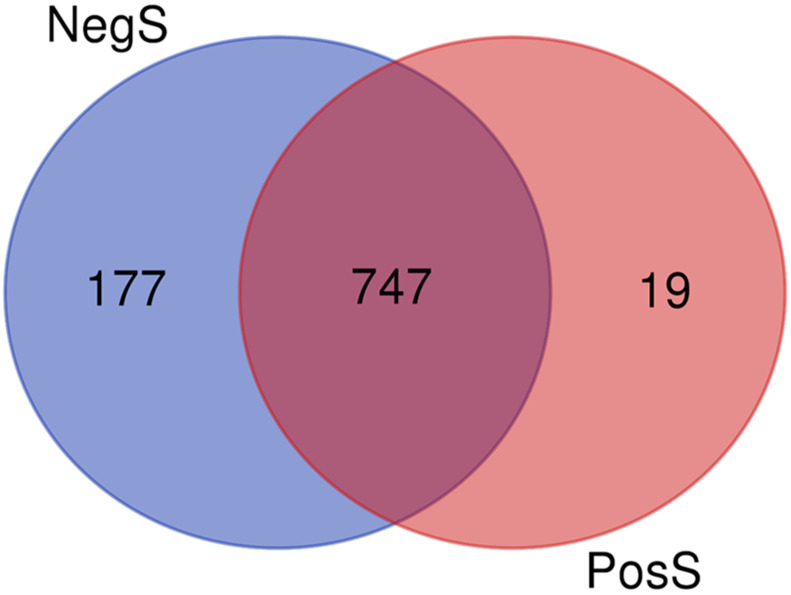
Venn diagram showing the distribution of 943 conserved miRNAs of both evaluated groups.

**Figure 6:**
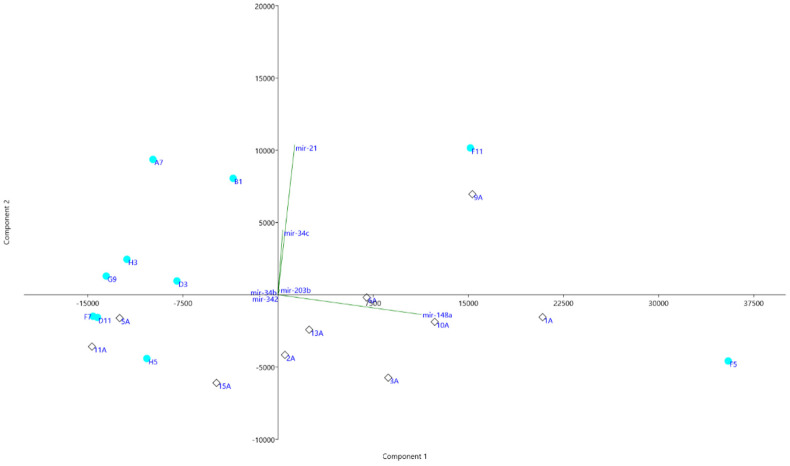
Principal component analysis (PCA) of the six miRNAs that distinguish the two groups. Black diamonds—NegS; aqua dots—PosS. miRNAs miR-21, miR-34c, miR-203b, miR-34b, miR-342, and miR-148a are included.

A total of 943 conserved miRNAs were identified in nasopharynx tissues (Supplementary Materials Tables S1 and S2). Among them, five conserved miRNAs were selected for detailed evaluation based on small RNA sequencing results (Figure 3 and Figure 4). The distribution of conserved miRNAs within the NegS and PosS groups is depicted in Figure 5 and Supplementary Materials Table S3. The most highly expressed miRNA in the NegS group was miR-148a, and the reads ranged from 3373 (sample 11A) to 38,424 (1A) with a median value of 22,341. The most highly expressed miRNA in the PosS group was miR-148, which ranged from 3209 (F7) to 53,305 reads (F5) with a median value of 7233 (Figure 6, Supplementary Materials Table S4).

**Figure 7:**
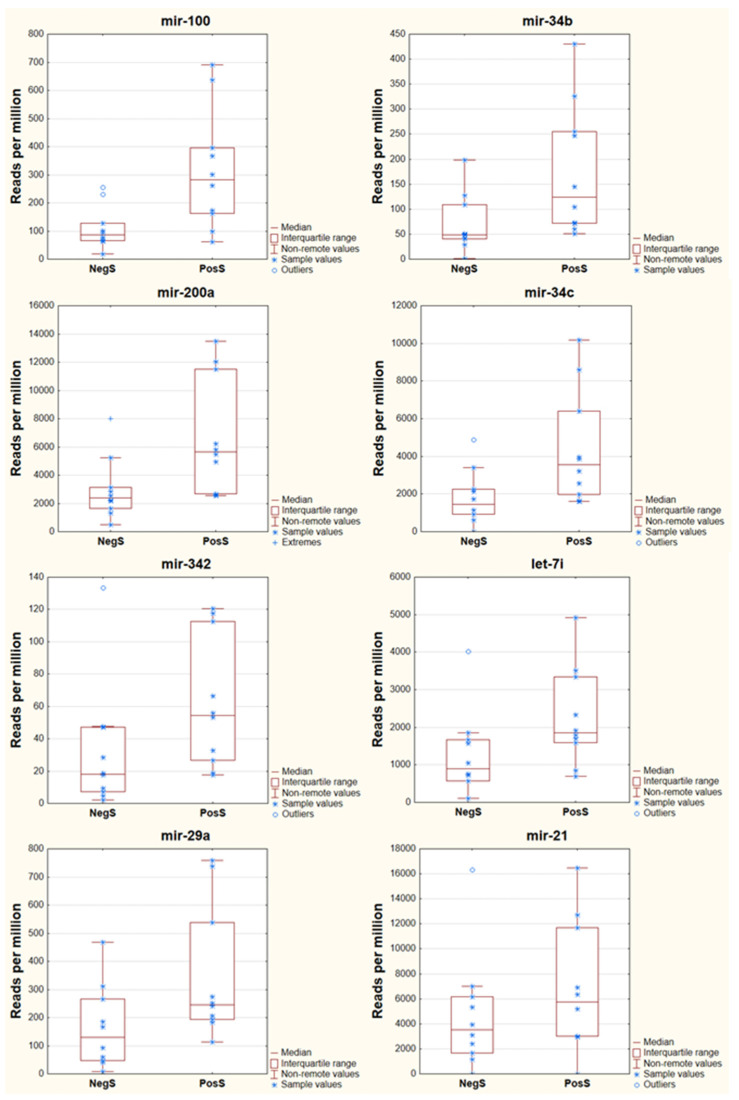
Normalized miRNA (RPM) expression of eight selected miRNAs between the NegS and PosS groups. Each selected miRNA showed significantly different expressions based on small RNA sequencing data, except the miR-21 (*p*-value 0.1306).

A test for normal distribution showed that the obtained numbers of reads did not fit a normal distribution of the residuals. We used a nonparametric method, the Kruskal–Wallis test, and the *p*-values of the most abundant miRNA datasets ranged from 0.0113 (miR-100) to 0.04125 (miR-29a). Statistically significant differences were determined between NegS and PosS for miR-100, miR-34b, miR-200a, miR-34c, mir-342, let-7i, and miR-29a (Table 3, Figure 7), where the miRNAs were upregulated in samples of the PosS group. The abundance of conserved mir-148a and mir-21 was not significantly different between NegS and PosS.

### 3.3. miRNA Expression Measured by Real-Time RT-PCR

**Figure 8:**
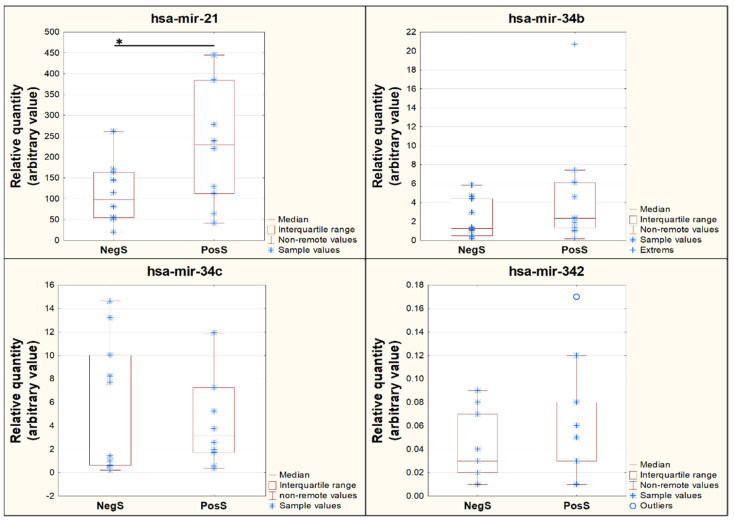
Normalized miRNA expression of four selected miRNAs between the NegS and PosS groups. Significantly different expressions (*p* ≤ 0.05) are indicated with a black asterisk.

Analysis of miRNA expressions normalized to mir-148a miRNA revealed that three miRNAs (miR-21, miR-34b, and miR-342) were upregulated in the PosS group (Table 4, Figure 8). Of these miRNAs, only miR-21 showed significantly higher expression in the PosS group than in the NegS group. One miRNA, miR-34c, was downregulated in the PosS group; however, there was no significant difference between the PosS and NegS groups in the expression of this miRNA (Table 4, Figure 8).

Correlation analysis revealed that the expressions of mir-21 and mir-342 were in strong and very strong, respectively, positive correlation with viral relative quantity (Table 4). The expressions of mir-34c and mir-34b showed only very weak and moderate, respectively, correlation with the viral relative quantity.

## 4. Discussion

This is the first attempt to use a small RNA high-throughput sequencing technique to identify conserved miRNAs differentially expressed in human nasopharynx tissues in response to SARS-CoV-2 infection. The experimental strategy of this study was designed to investigate the profile of human miRNAs in two groups with 10 samples that tested negative (NegS) and positive (PosS) for SARS-CoV-2 by real-time RT-PCR. Human–pathogen interactions were completely evaluated at the level of total extracted RNA with no additional information concerning the examined individuals. miRNA profiling and prediction associated with SARS-CoV-2 in positive human RNA was performed in previous studies by Arisan et al. [23], Chen et al. [24], Chow and Salmena [25], Demongeot and Seligman [26], Hosseini Rad and McLellan [27], and Sardar et al. [28]; however, these studies used theoretical prediction based on the available datasets without performing practical sequencing experiments. The study of Li et al. [29] first used a high-throughput sequencing approach for profiling the miRNAs in the peripheral blood from patients with SARS-CoV-2 infection. Lu et al. [30] used the real-time RT-PCR method to quantify SARS-CoV-2-associated miRNAs in mouse cardiomyocytes targeting the SARS-CoV-2 entry receptor ACE2.

Based on the hypothesis that miRNAs associated with SARS-CoV-2 infection are present in nasopharynx tissues, we identified 943 conserved miRNAs. We revealed that the most abundant small RNAs were the 21-(PosS) and 22-nt classes (NegS). Fang et al. [31] mentioned that the majority of mature human miRNA sequences consist of 22 nucleotides. We revealed that the reads of the 22-nt miRNA class were not normally distributed among NegS and PosS, and the values were not significantly different (*p*-value 0.7624). The reads of the 21-nt class did not show any significant difference, similar to the 22-nt class (*p*-value 0.4057). The overexpression of 21-nt miRNA class reads in PosS could be associated with the disease course. According to published results, duplexes of 21 nucleotide short interference RNA (siRNA) with 2-nucleotide in the 3′-overhangs are the most efficient triggers of nucleotide sequence-specific mRNA degradation [32,33].

According to the differences in abundances between the NegS and PosS groups, we focused on the five following conserved miRNAs: miR-148a, miR-21, miR-34c, miR-34b, and miR-342. The most abundant miRNA was miR-148a in the NegS group and in the PosS group, with no significant difference, but a higher expression was observed in the NegS group. MiR-148a in human tissues plays a key role in many biological processes. MiR-148a has ordinary functions shared by many miRNA classes, including a role in cellular differentiation and development. Porstner et al. [34] proved that miR-148a expression increased after pre-B cell activation. Moreover, upregulated miR-148a expression helps to the differentiation of activated B cells to plasma cells and so it helps the survival of plasma cells by constraining various transcription and proapoptotic factors. This miRNA is a muscle-derived miRNA that may facilitate the differentiation of myoblasts and skeletal muscle cells by targeting regulatory Rho-associated coiled-coil containing protein kinase 1. MiR-148a may also promote the growth of myoblasts in the G1 phase of the cell cycle and shorten the S phase. This role prompts myoblast differentiation into myotubes [35]. Van Wijnen et al. [36] showed that miR-148a is involved in osteoclast formation. MiR-148a induces the transformation of monocytes to osteoclasts by inhibition of the transcription factor V-maf musculoaponeurotic fibrosarcoma oncogene homolog B. Gailhouste et al. [37] showed that enhanced expression of miR-148a might induce the differentiation and maturation of liver cells by inhibiting DNA. MiR-148a also can promote primary adipocytes to differentiate into mature adipocytes [38]. MiR-148a may affect the development of the nervous system by targeted regulation too [39]. It has been shown that miR-148a regulates several phenotypes, including those present in embryonic stem cells [40]. Downregulated expression of miR-148a can control the phenotype of mesenchymal stem cells. This is done by helping the expression of the endothelial PAS domain that contains protein 1 transforming factor [41]. The downregulated expression of miR-148a can be detected in many types of cancers, including gastric, colorectal, pancreatic, liver, oesophageal, breast, non-small cell lung, and urogenital system cancers. Nonetheless, upregulated expression of miR-148a may be seen also in glioma and osteosarcoma. Moreover, the expression levels of miR-148a have been clearly and strongly linked to the clinical classification, efficacy, and prognosis of cancer [42].

Second, the most abundant miRNA in both investigated groups was miR-21. miR-21 was significantly differentially expressed between the NegS and PosS groups according to real-time RT-PCR (*p*-value 0.03156). In contrast to miR-148, based on the results miR-21 probably has a direct link to SARS-CoV-2 infection. No significant difference (*p*-value 0.1306) was observed between the NegS and PosS groups by small RNA sequencing, but higher expression of miR-21 was observed in the PosS group according to both detection methods (Table 3 and Table 4, Figure 4, Figure 5 and Figure 6). The scatter plot (Figure 6) also showed that higher abundances of miR-21 were typical for the PosS group. This means that the trend is the same as was found out by real-time RT-PCR. Moreover, a strong positive correlation between miR-21 expression and COVID-19 titer (Pearson’s correlation coefficient 0.73, Table 4) indicates a tight connection between miR-21 expression and infection with COVID-19. Farr et al. [43] have recently posted the same results, showing that miR-21 was upregulated in COVID-19. miR-21, a SARS-CoV-2-binding microRNA, has confirmed four binding sites on the SARS-CoV-2 genome. mir-21 is one of the better-known miRNAs whose expression increases in many pathological conditions, including asthma, pulmonary fibrosis, and viral infection [44,45]. There are no reports on the direct binding of miR-21 to other human viral genomes in recent years, and current reports about the involvement of miR-21 in viral infections are very limited to host transcripts modulating. For example, the positive role of miR-21 in influenza A replication was attributed to the miR-21-host HDAC8 interaction [46]. In addition, it has been shown that miR-21 reduces the antiviral NF-KB pathway by binding to IRAK1 and TRAF6 transcripts in HIV and HCV infections [47,48]. According to the results of Jafarinejad-Farsangi et al. [49], miR-21 has two binding sites in the spike protein-coding regions. In addition, miR-21 was one of the top miRNAs that targeted the upregulated host DEGs in response to SARS-CoV-2, which agrees with our results, and it showed normalized median reads of NegS (3531) versus PosS (5772). One of the miR-21 targets is CXCL-10, which is a biomarker for viral, bacterial, fungal, and parasitic contamination [50]. In the study of Jafarinejad-Farsangi et al. [49], high levels of CXCL-10 were observed in the lungs of COVID-19-infected patients compared to healthy patients. miR-21 expression was increased in the COVID-19 group compared to healthy controls [51].

The rest of the comprehensively quantified miRNAs in this study showed significant differences between the NegS and PosS groups according to small RNA sequencing. The third most expressed miRNA was mir-34c. The number of normalized miR-34c reads was significantly different according to a *p*-value of 0.02334, and miR-34c was upregulated in PosS (3350 reads) compared to NegS (1444 reads); however, this phenomenon was not confirmed by real-time RT-PCR. This is only one miRNA where the trend is different between small RNA sequencing and real-time RT-PCR results. The results of small RNA sequencing are in contrast with a case-control study [52] in which respiratory syncytial virus (family *Paramyxoviridae*)-infected patients showed low levels of miR-34c expression compared with controls. qPCR is often considered a gold standard in the detection and quantitation of various gene expressions. Nevertheless, the rapid increase in the number of miRNAs renders qPCR inefficient on a genomic scale, and it is probably better used as a validation rather than a discovery tool [53]. Small RNA sequencing comes into wider use and is unmatched for the discovery and experimental validation of novel or predicted miRNAs [53]. miR-34c is a 77 bp long noncoding RNA. This miRNA is located on human chromosome 11 belonging to the miR-34 family. The miR-34 family may regulate cell processes by binding to target gene sequence fragments. miR-34c inhibits Bcl2 by binding to the 3′ untranslated region (UTR) of the Bcl2 gene, thus downregulating the viability of laryngeal cancer cells and inducing apoptosis [54]. miR-34c is also associated with emphysema severity and thus modulates SERPINE1 expression [55]. SERPINE1 encodes a member of the serine proteinase inhibitor superfamily. SERPINE1 is the principal inhibitor of tissue plasminogen activator and urokinase. The final protein also functions as a component of innate antiviral immunity and high concentrations of the gene product are associated with thrombophilia [56].

miR-34b was detected by small RNA sequencing by 49 reads in the NegS group and by 124 reads in the PosS group, and the *p*-value was 0.01258. The results were confirmed by real-time RT-PCR but not with significant values (*p*-value 0.22542, Pearson’s correlation coefficient 0.48). In total, we determined seven miRNAs with statistically significant expression level differences (miR-100, miR-34b, miR-200a, miR-34c, miR-342, let-7i, and miR-29a). The results are not in line with the current study by Demiray et al. [57]. Statistically significant expression level differences (*p*  <  0.05) were detected by real-time RT-PCR in nine miRNAs in COVID-19 patients and healthy controls. Seven miRNA (let-7d, miR-17, miR-34b, miR-93, miR-200b, miR-200c, and miR-223) expression levels were found to be significantly decreased, and the expression levels of two miRNAs (miR-190a and miR-203) were significantly increased compared to healthy controls [57]. In total, the findings that have been captured by Demiray et al. [57] can be validated by our experiment only in the case of miR-203 and miRNAs let-7d, miR-17, miR-34b, miR-93, miR-200b, miR-200c, miR-223, and miR-190a showed the opposite expression. Moreover, it is not clear why Demiray et al. [57] focused on 21 miRNAs expressed in serum and how selected these 21 miRNAs.

The lowest level of expression of the selected miRNAs was observed for miR-342; in the NegS group, it was detected by 18 reads, and in the PosS group, it was detected by 55 reads (*p*-value 0.0411). Its upregulation was also observed by real-time RT-PCR, and the relative expression values were 0.042 (NegS) and 0.065 (PosS group) with a significance *p*-value of 0.24653. Interestingly, there was a very strong positive correlation between miR-342 expression and the COVID-19 titer (Pearson’s correlation coefficient 0.87, Table 4). However, the correlation result may not be relevant as, although significantly upregulated in the PosS group unveiled by small RNA sequencing, the miR-342 expression did not differ in PosS and NegS in real-time RT-PCR. The computational approach revealed an exciting hypothesis that miR-342 is involved in MERS-CoV pathogenesis [58]. mir-342-5p also suppresses coxsackievirus B3 biosynthesis by targeting the 2C-coding region [59].

The identified miRNAs were expressed in both RNA groups, NegS and PosS: miR-148a, miR-21, miR-34c, miR-34b, and miR-342. The most differentially expressed miRNA was miR-21, which is likely closely linked to the presence of SARS-CoV-2 in nasopharynx tissues. Upregulation of miR-21 in the PosS group was almost two times higher and could also be linked with cardiac fibroblast and endothelial cell dysfunction in COVID-19 patients [51]. Due to the nature of our results, it is probable that a rapid increase in the number of miRNAs renders qPCR inefficient on a genomic scale, and it is maybe better to use small RNA sequencing as a discovery tool. To our knowledge, this is the first study reporting important miRNAs detected in SARS-CoV-2-positive RNA extracted from nasopharyngeal tissues. Although this study encompasses the role of RNAs, a description of the symptoms caused by SARS-CoV-2 is missing and future studies should include this information. These findings should serve as a foundation for larger studies and should contribute to evaluating the long-term course of COVID-19 patients.

## Figures and Tables

**Table 1:** List of the RNA samples. Ct values were generated with the E gene assay [18]. The relative quantity of the virus was generated with the E gene results normalized to hsa-mir-148a miRNA.

Negs Group	Ct (Threshold Cycle)	Poss Group	Ct (Threshold Cycle)	Relative Quantity (Arbitrary Values)
1A	>40	D3	31.90	87.2
2A	>40	H3	27.68	5245.9
3A	>40	H5	32.51	92.9
5A	>40	F5	33.75	2.2
6A	>40	A7	25.83	185,29.1
9A	>40	B1	29.52	18.3
10A	>40	G9	24.82	121,61.2
11A	>40	F11	33.04	4.6
13A	>40	D11	26.50	122,20.1
15A	>40	F7	32.00	29.5

**Table 2:** Numbers of size distributions of unique reads normalized to reads per 1,000,000. The length of detected small RNAs was 19–25 nucleotides.

Group	Sample	19	20	21	22	23	24	25	Total
**Group NegS**	1A	4215	6687	13,399	18,965	8843	1624	125	53,858
	2A	574	1062	2058	3768	1502	907	224	10,095
	3A	657	1172	2068	4796	1784	755	104	11,336
	5A	1271	2164	3701	4485	1707	476	49	13,853
	6A	2909	5335	9794	10,830	4356	835	57	34,116
	9A	4552	10,212	19,191	17,643	8898	2746	298	63,540
	10A	3370	6228	22,709	50,524	11,225	3031	286	97,373
	11A	891	1601	3247	2211	817	146	16	8929
	13A	2589	3674	8894	8365	3924	774	101	28,321
	15A	2679	5458	8792	11,024	5776	947	71	34,747
	Median	2634	4505	8843	9598	4140	871	103	31,219
	Average	2371	4359	9385	13,261	4883	1224	133	35,617
**Group PosS**	D3	1936	4308	6113	6331	3895	1174	173	23,930
	H3	2459	5712	7016	5567	2308	852	96	24,010
	H5	2073	3081	5488	5702	2676	778	146	19,944
	F5	3832	6566	10,998	12,974	8704	1704	141	44,919
	A7	6065	12,807	48,308	26,818	5361	1182	100	100,641
	B1	6225	13,122	48,366	30,578	5131	903	120	104,445
	G9	2696	4525	5930	6959	3343	1041	74	24,568
	F11	7574	12,936	23,790	23,718	12,065	2358	165	82,606
	D11	2569	3715	5992	4420	1286	274	25	18,281
	F7	1097	2472	3949	3922	1832	476	57	13,805
	Median	2569	4525	6113	6331	3343	903	100	24,289
	Average	3322	6297	15,088	11,546	4239	979	102	45,715

**Table 3:** Kruskal–Wallis test emphasizing statistically significant differences between the NegS and PosS groups of five selected miRNAs. All miRNA sequences with total abundance >2000 reads are included. The normalized read (RPM) abundances are listed. Significantly different expressions (* *p* ≤ 0.05) are indicated with an asterisk.

miRNA	*p*-Value	NegS (Median)	PosS (Median)
mir-100	0.0113 *	86	282
mir-34b	0.01258 *	49	124
mir-200a	0.01556 *	2406	5631
mir-34c	0.02334 *	1444	3550
mir-342	0.0411 *	18	55
let-7i	0.04125 *	903	1856
mir-29a	0.04125 *	130	246
mir-141	0.06954	100	153
mir-222	0.06954	99	148
mir-200c	0.06964	672	1152
mir-146a	0.0821	231	416
let-7f	0.1124	252	592
mir-21	0.1306	3531	5772
let-7a	0.1509	193	410
mir-203b	0.1736	122	300
mir-99a	0.1857	1327	2536
mir-25	0.1986	141	195
mir-183	0.1986	207	102
mir-200b	0.1988	1656	2222
let-7c	0.2265	251	532
mir-1301	0.239	7	9
mir-146b	0.2568	150	316
mir-22	0.2896	269	290
mir-148a	0.2899	22341	7233
let-7b	0.2899	1296	2747
mir-320a	0.2899	145	213
mir-26b	0.2899	115	164
mir-429	0.4497	263	263
mir-423	0.4629	368	467
mir-26a	0.4963	292	532
mir-20b	0.5182	0	0
mir-30a	0.5453	240	248
mir-92b	0.5453	244	251
mir-3960	0.623	167	76
mir-99b	0.677	82	110
let-7g	0.6964	469	771
mir-3074	0.7054	472	522
mir-27a	0.7055	1326	1027
mir-30d	0.7055	860	672
mir-27b	0.7624	1264	1072
mir-205	0.7624	252	157
mir-375	0.8205	797	687
mir-151a	0.8206	1627	1523
mir-10400	0.9097	171	82

**Table 4:** Normalized miRNA expression measured by real-time RT-PCR between the NegS and PosS groups of four selected miRNAs and correlation determination between normalized miRNA expressions and relative quantities of viral titer. Significantly different expressions (* *p* ≤ 0.05) are indicated with an asterisk.

miRNA	*p*-Value	Negs (Mean)	Poss (Mean)	Pearson’s Correlation Coefficient
mir-21	0.03156 *	111.56	235.61	0.73
mir-34c	0.49070	5.76	4.26	0.15
mir-34b	0.22542	2.25	4.79	0.48
mir-342	0.24653	0.042	0.065	0.87

## Data Availability

Not applicable.

## Supplementary Materials

The following supporting information can be downloaded at: https://www.mdpi.com/article/10.3390/genes13020348/s1, Tables S1 and S2: A total of conserved miRNAs identified in nasopharynx tissues, Table S3: The distribution of conserved miRNAs within the NegS and PosS groups (RPM), Table S4: The most highly expressed miRNA in the PosS group.
