# Supplementary material for: Conserved MicroRNAs in Human Nasopharynx Tissue Samples from Swabs Are Differentially Expressed in Response to SARS-CoV-2
Source: Genes (Basel). 2022 Feb 14;13(2):348. doi: 10.3390/genes13020348 (PMC8871708; doi:10.3390/genes13020348)
Supplement: Supplementary file 1 [file genes-13-00348-s001.zip › Figure legends.docx]

Figure legends

Fig. 1 Bar plot depicting the size distribution of unique reads in the 19- to 25-nt class of sRNAs in the NegS group.

Fig. 2 Bar plot depicting the size distribution of unique reads in the 19- to 25-nt class of sRNAs in the PosS group.

Fig. 3 Bar plot depicting the proportions of five known selected human mature miRNAs according to their different abundances in both groups across NegS, CLC Genomics Workbench 6.5.1, and miRBase Release 22.1. Proportions were calculated based on normalized total reads.

Fig. 4 Bar plot depicting the proportions of known selected human mature miRNAs according to their different abundances in both groups across PosS, CLC Genomics Workbench 6.5.1, and miRBase Release 22.1. Proportions were calculated based on normalized total reads.

Fig. 5 Venn diagram showing the distribution of 943 conserved miRNAs of both evaluated groups.

Fig. 6 Normalized miRNA expression of four selected miRNAs between the NegS and PosS groups. Significantly different expressions (p ≤ 0.05) are indicated with an asterisk. SE means standard error.

Fig. 6 Normalized miRNA expression of four selected miRNAs between the NegS and PosS groups. Significantly different expressions (p ≤ 0.05) are indicated with an asterisk. SE means standard error.
