# Supplementary material for: Conserved MicroRNAs in Human Nasopharynx Tissue Samples from Swabs Are Differentially Expressed in Response to SARS-CoV-2
Source: Genes (Basel). 2022 Feb 14;13(2):348. doi: 10.3390/genes13020348 (PMC8871708; doi:10.3390/genes13020348)
Supplement: Supplementary file 1 [file genes-13-00348-s001.zip › ZDPM89FB_024B-94E0-945B-E7D2-E70A.pdf]

AJE®

Editing Certificate

This document certifies that the manuscript

Conserved microRNAs in human nasopharynx tissue samples from swabs are differentially expressed in response to SARS-CoV-2

prepared by the authors

Aleš Eichmeier. Tomáš Kiss. Mária Kocanová. Eliška Hakalová. Milan Špetík. Jana Čechová. Boris Tichý

was edited for proper English language, grammar, punctuation, spelling, and overall style by one or more of the highly qualified native English speaking editors at AJE.

This certificate was issued on **July 20, 2021** and may be verified on the AJE website using the verification code **024B-94E0-945B-E7D2-E70A** .

Neither the research content nor the authors' intentions were altered in any way during the editing process. Documents receiving this certification should be English-ready for publication; however, the author has the ability to accept or reject our suggestions and changes. To verify the final AJE edited version, please visit our verification page at aje.com/certificate. If you have any questions or concerns about this edited document, please contact AJE at support@aje.com.

AJE provides a range of editing, translation, and manuscript services for researchers and publishers around the world. For more information about our company, services, and partner discounts, please visit aje.com.
