# Supplementary figures and images for: Conserved MicroRNAs in Human Nasopharynx Tissue Samples from Swabs Are Differentially Expressed in Response to SARS-CoV-2
Source: Genes (Basel). 2022 Feb 14;13(2):348. doi: 10.3390/genes13020348 (PMC8871708; doi:10.3390/genes13020348)

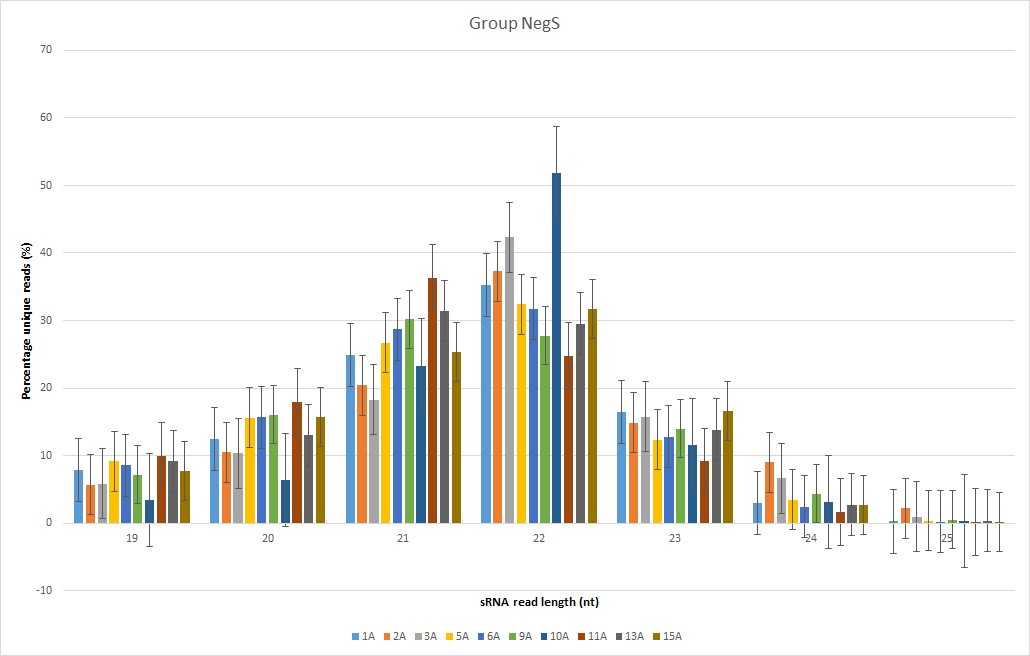

Supplement: Supplementary file 1 [file genes-13-00348-s001.zip › Fig_1.tif]

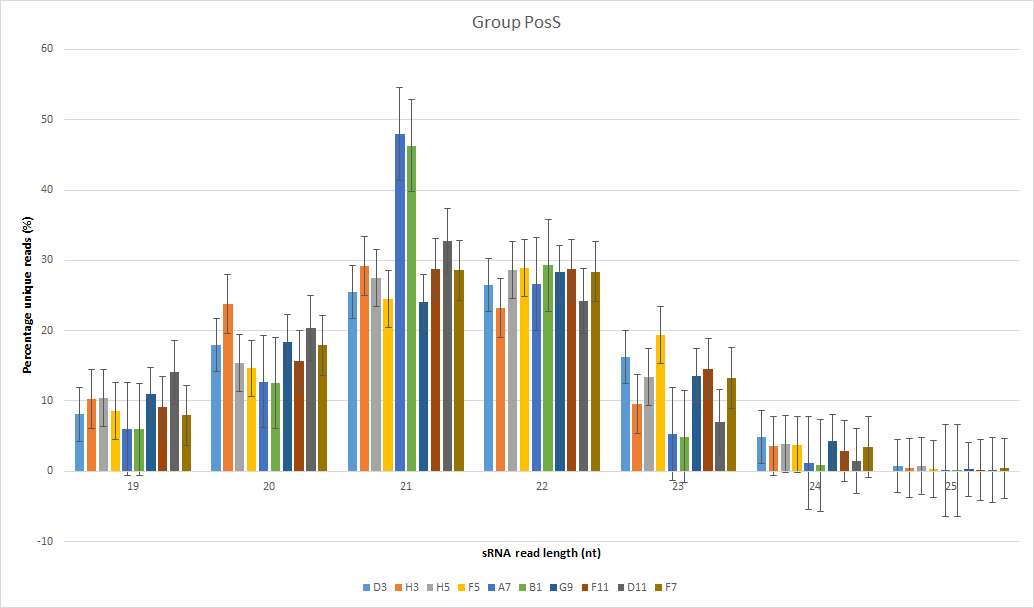

Supplement: Supplementary file 1 [file genes-13-00348-s001.zip › Fig_2.tif]

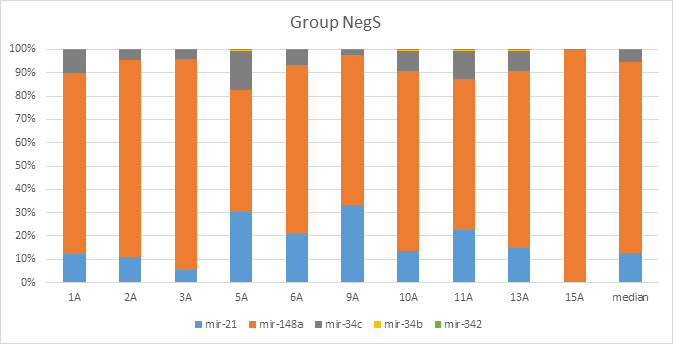

Supplement: Supplementary file 1 [file genes-13-00348-s001.zip › Fig_3.tif]

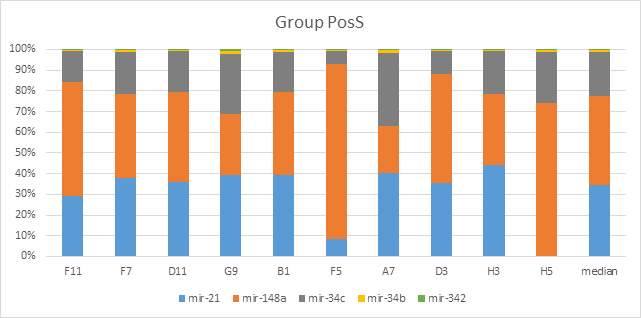

Supplement: Supplementary file 1 [file genes-13-00348-s001.zip › Fig_4.tif]

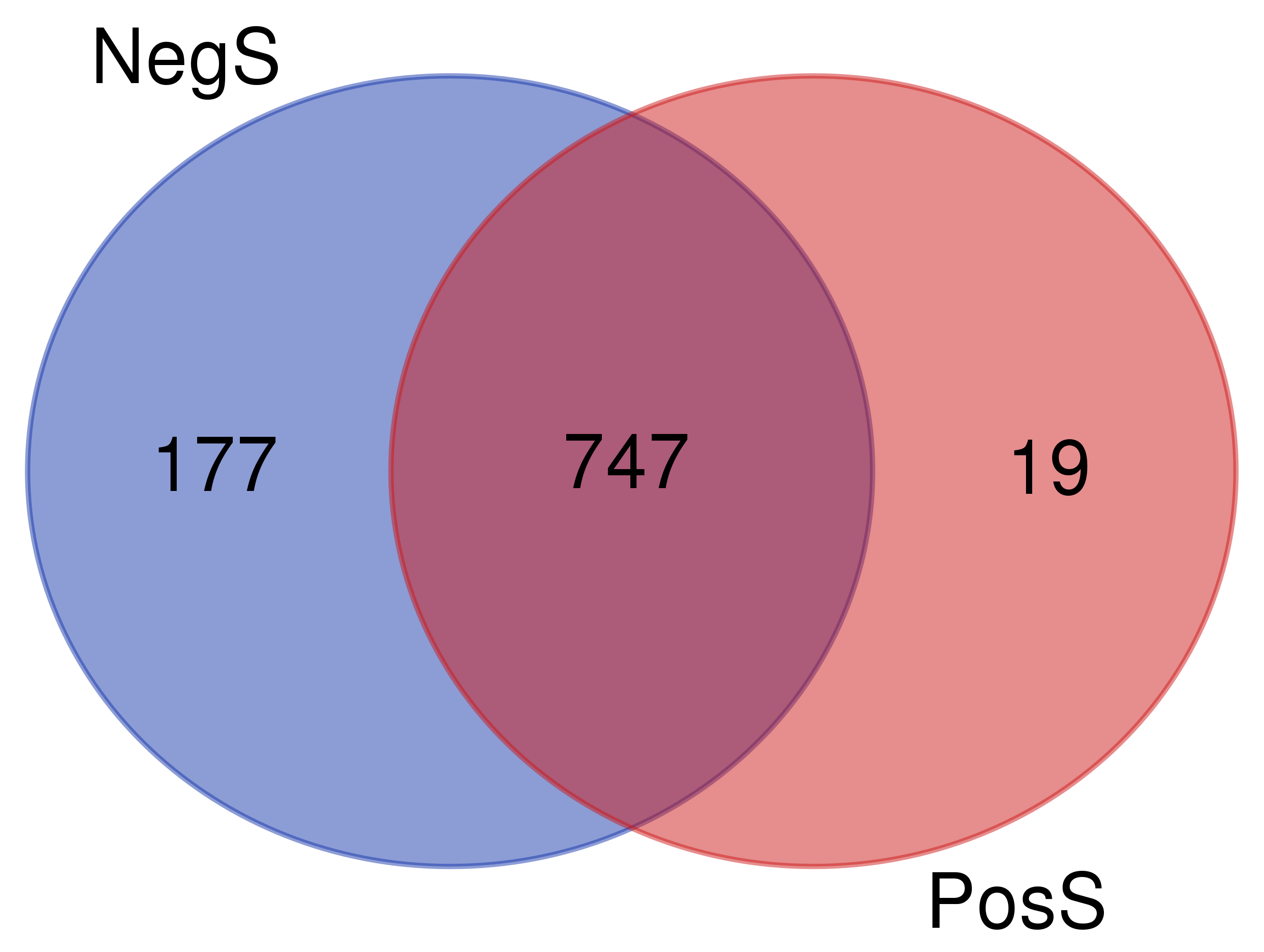

Supplement: Supplementary file 1 [file genes-13-00348-s001.zip › Fig_5.tif]

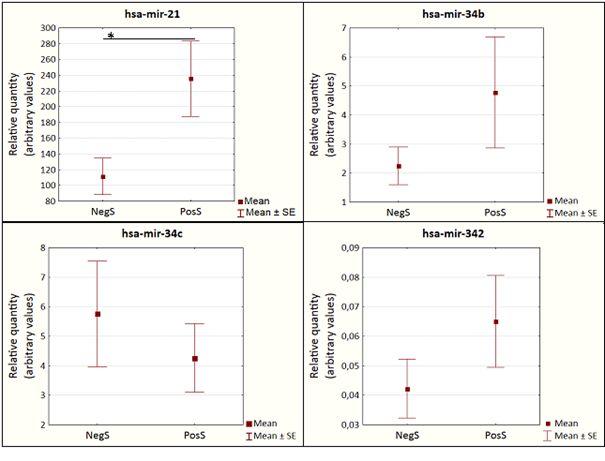

Supplement: Supplementary file 1 [file genes-13-00348-s001.zip › Fig_6.tif]
